# First report of potato rot nematode, *Ditylenchus destructor* Thorne, 1945 infecting *Codonopsis pilosula* in Gansu province, China

**DOI:** 10.21307/jofnem-2020-087

**Published:** 2020-08-31

**Authors:** Chunhui Ni, Shuling Zhang, Huixia Li, Yonggang Liu, Wenhao Li, Xuefen Xu, Zhipeng Xu

**Affiliations:** 1 College of Plant Protection, Gansu Agricultural University/Biocontrol Engineering Laboratory of Crop Diseases and Pests of Gansu Province, Lanzhou, 730070, Gansu Province, China; 2 Institute of Plant Protection, Gansu Academy of Agricultural Sciences, Lanzhou, 730070, Gansu Province, China

**Keywords:** Chinese herbal medicine, *Ditylenchus destructor*, Molecular biology, Morphology, New host

## Abstract

In November 2019, stem nematode was found on *Codonopsis pilosula* in Tanchang county, Gansu province, China. The population of stem nematode was identified on the basis of both molecular and morphological methods. The morphological and morphometric characteristics of this nematode population matched with *Ditylenchus destructor* Thorne, 1945. The sequences of rDNA-ITS and D2/D3 region of 28S-rRNA similarity with the *D. destructor*. The pathogenicity results revealed the symptom of dry rot on *C. pilosula* was caused by this nematode. To our knowledge, this is the first report that *D. destructor* on *C. pilosula* in China.

The roots of diseased plants were collected and nematodes were extracted using a modified Baermann technique (Hooper, 1990). The results of morphological and morphometric characteristics of this nematode population were as following. Lip regions were plain with obscure constriction and stylets are tiny with 9.9 to 10.8 (μm) long and distinct knobs. Oval median bulbs were with valves, narrow isthmus and the posterior esophageal extended over intestines from the dorsad. Lateral fields marked by six incisures and tail tips were rounded. The vulvas of females at the back of the body were slightly protruding and the posterior uterine sac extended to the anus, which was about 3/4 of the distance from the vulva to the anus. Male bodies were similar to those of females and slightly bent spicules were strong with bursas encircling to 1/3 of the tail.

The morphometrics (mean ± SD) of the nematodes were as following. Females (*n* = 20): *L* = 980.8 ± 182.5 (779.1-1,131.2) μm, *a* = 39.7 ± 6.5 (33.6-49.2), *b* = 6.7 ± 1.0 (5.3-7.6), *c* = 15.6 ± 2.0 (13.3-18.5), *c*′ = 3.8 ± 0.3 (3.4-4.2), *V* = 81.3 ± 2.4 (77.8-83.9), *V*′ = 106.9 ± 0.9 (105.7-108.1), stylet length: 11.3 ± 0.9(9.8-12.3) μm, tail length: 62.8 ± 9.2 (55.6-78.5) μm, ABW = 16.5 ± 2.5 (13.4-20.1) μm.

Males (*n* = 20): *L* = 772.0 ± 92.8 (679.8-876.6) μm, *a* = 40.3 ± 2.8 (37.3-43.0), *b* = 5.4 ± 0.4 (4.9-6.0), *c* = 12.8 ± 1.1 (11.5-14.5), *c*′ = 4.1 ± 0.3 (3.7-4.5), stylet length: 10.3 ± 0.4 (9.9-10.8) μm, tail length: 60.2 ± 5.0 (55.5-67.7) μm, ABW = 16.5 ± 2.5 (13.4-20.1) μm. These morphological characteristics matched with *Ditylenchus destructor* (Thorne, 1945).

DNA of single nematode (*n* = 5) was isolated using the Proteinase K method (Kumari and Subbotin, 2012), and amplification of rDNA-ITS region and sequencing were performed with the universal primers 18S (5′-TTG ATT ACG TCC CTG CCC TTT-3′) and 26S (5′-TTT CAC TCG CCG TTA CTA AGG-3′) (Vrain et al., 1992). The sequence of rDNA-ITS (978 bp; MT150860, MT150861) were submitted to GenBank, and the BLAST result showed that these sequences were 99.90% identical to the *D. destructor* on potato from Hebei Province in China (FJ911551) and on sweet potato from Shandong Province in China (EF208212). Therefore, the nematode population was identified as *D. destructor*.

To confirm the pathogenicity of the population, the healthy *C. pilosula* seedlings which were sterilized with alcohol (75%) and NaClO (2.5%) were planted into sterilized substrates in a greenhouse. After two weeks, every plant was inoculated with about 5,000 *D. destructor* near roots, repeated five plants and three plants served as control. After 60 days, symptoms on *C. pilosula* similar to those in the field were observed and *D. destructor* was isolated from inoculated plants. The control plants remained healthy. The results revealed the symptom of dry rot on *C. pilosula* was caused by this nematode. To our knowledge, this is the first report that *D. destructor* could infect *C. pilosula* and *C. pilosula* is a new host of it. By now, *D. destructor* damaged on angelica and potato in Gansu province (Wang et al., 1990; Li et al., 2016). Since *C. pilosula* is an important cash crop in Gansu province, more attentions should be paid to *D. destructor* on *C. pilosula*.AcknowledgmentsThis research was supported by the National Natural Science Foundation of China (31760507) and National Key Research and Development Plan (2018YFC1706301).
